# Local public health response to vaccine-associated measles: case report

**DOI:** 10.1186/1471-2458-13-269

**Published:** 2013-03-25

**Authors:** Monica Hau, Kevin L Schwartz, Crystal Frenette, Isabelle Mogck, Jonathan B Gubbay, Alberto Severini, Joanne Hiebert, Shelley L Deeks, Shaun K Morris

**Affiliations:** 1 Peel Public Health, 7120 Hurontario St. RPO Streetsville, P.O Box 667, Mississauga, ON, L5M 2C2, Canada; 2 Dalla Lana School of Public Health, University of Toronto, 155 College St, 6th floor, Toronto, ON, M5T 3M7, Canada; 3 Department of Pediatrics, Faculty of Medicine, University of Toronto, 1 King’s College Circle, Toronto, ON, M5S 1A8, Canada; 4 Division of Infectious Diseases, The Hospital for Sick Children, 555 University Avenue, Toronto, ON, M5G 1X8, Canada; 5 Public Health Ontario, 480 University Avenue, Suite 300, Toronto, ON, M5G 1V2, Canada; 6 National Microbiology Laboratory, Public Health Agency of Canada, 1015 Arlington Street, Winnipeg, MB, R3E 3E2, Canada; 7 Department of Microbiology and Infectious Diseases, Faculty of Medicine, University of Manitoba, Rm 543 – 745, Bannatyne Avenue, Winnipeg, MB, R3E 0J9, Canada

**Keywords:** (3–10): measles, Immunization, Vaccine associated, Vaccine strain, Contact tracing, Adverse event

## Abstract

**Background:**

The most appropriate public health approach to vaccine-associated measles in immunocompromised patients is unknown, mainly because these cases are rare and transmission of vaccine-associated measles has not been previously documented. In this case report, we describe Peel Public Health’s response to a vaccine-associated measles case in an immunocompromised child in Ontario, Canada.

**Case presentation:**

A five-year-old Canadian-born boy with a history of a hematopoetic stem cell transplant three years previously received live attenuated measles, mumps, and rubella (MMR) vaccine. Over the subsequent 7 to 14 days, he developed an illness clinically consistent with measles. There was no travel history or other measles exposure. Serology and polymerase chain reaction (PCR) testing confirmed acute measles infection. Following discussion with pediatric infectious diseases specialists, but prior to the availability of virus sequencing, it was felt that this case was most likely due to vaccine strain. Although no microbiologically confirmed secondary cases of vaccine-associated measles have been previously described, we sent notification letters to advise all contacts of measles symptoms since the likelihood of transmission from an immunocompromised patient was low, but theoretically possible. We decided to stratify contacts into immune competent and compromised and to deal with the latter group conservatively by excluding them as if they were exposed to wild-type measles because the risk of transmission of disease in this population, while presumably very low, is unknown. However, no contacts self-identified as immunocompromised and there were no secondary cases. Subsequent genotyping confirmed that this case was caused by vaccine strain measles virus.

**Conclusion:**

The public health approach to contact tracing and exclusions for vaccine-associated measles in immunocompromised patients is unclear. The rarity of secondary cases provides further evidence that the risk to the general public is likely extremely low. Although the risk appears negligible, exclusion and administration of immune globulin may be considered for susceptible, immunocompromised contacts of cases of vaccine-associated measles in immunocompromised patients.

## Background

While the public health approach to wild-type measles infection in terms of contact tracing, isolation, and post exposure immunization or immune globulin is well described [1], the most appropriate course of action in cases of vaccine-associated measles in immunocompromised individuals is not clear. Vaccine-associated measles infections that are clinically indistinguishable from wild-type measles are rare and have occurred in both healthy and immunocompromised children [2-6]. Vaccine-associated measles has not been shown to be infectious, regardless of the immune status of the index case. Although studies have detected measles vaccine virus in pharyngeal and urine samples in immune competent children with a vaccine-associated febrile rash illness [3,7], to our knowledge, no microbiologically confirmed transmission to contacts has been documented. It is unknown, however, if vaccine-associated measles in immunocompromised cases changes the potential transmission risk to others. In this report, we describe a case of vaccine-associated measles in an immunocompromised child and the decisions made by Peel Public Health regarding contact tracing and the exclusion of susceptible, immunocompromised contacts.

## Case presentation

### Case description

A five-year-old Canadian-born boy who received a hematopoetic stem cell transplant (HSCT) at two years of age and received a dose of live attenuated measles, mumps, and rubella (MMR) vaccine (Schwartz – GSK) approximately three years post HSCT. There was no history of measles exposure nor travel, however by the 6^th^ day post vaccination, he had developed fever, cough, coryza, and conjunctivitis. On the 9^th^ day post vaccination he developed a maculopapular rash and on day 14 he was admitted to hospital with a clinical diagnosis of presumed measles. Measles IgM and IgG serology, taken one day after the onset of the rash, was initially negative. Subsequently, five days after rash onset, measles IgM and IgG became positive. In addition, measles real-time reverse transcriptase polymerase chain reaction (rRT-PCR) testing using an in-house assay targeting the fusion (F1), hemagglutinin (H1) and nucleoprotein (N3) genes was positive from nasopharyngeal (NP) swab, throat swab, and urine [8]. The virus was sequenced using the World Health Organization standardized method [9] and revealed measles genotype A, sequence designation MVs/Ontario.CAN/08.12 [A] (VAC), which is a vaccine strain. Viral cultures from the NP swab and urine were negative and the throat swab grew HSV-1. 

### Contact tracing and exclusion

Measles is a nationally reportable disease in Canada and health professionals are legally obligated to report measles to the local public health department [10]. Peel Public Health, in Peel Region and located directly west of Toronto, received a notification from the provincial reference laboratory about a measles PCR positive nasopharyngeal swab, throat swab, and urine sample, as well as positive IgM and IgG measles serology collected on the same day. After this notification, details were collected from a Toronto Public Health nurse regarding symptom onset and risk factors for measles because the case was hospitalized in Toronto. Given that the standard provincial case definition excludes those who have been recently vaccinated, it was initially not clear that case management and contact tracing should be pursued [11]. In addition, this patient did not have any epidemiologic links to other measles cases. However, we initiated an investigation since the case was symptomatic and we could not confidently rule out wild-type measles based on the initial serologic and PCR testing.

Several potential exposure sites where the child had attended during the infectious period were identified including two physician offices, one emergency department, a school, an after school playgroup and a school bus. These exposure sites included 617 contacts identified from the catchment area of six public health units. The immune status of the child was not clear due to his clinical presentation of measles post-vaccination, despite a previously normal immunological work-up details in a separate case report. As a result the pediatric infectious disease specialist advised that the usual period of communicability (four days before to four days after rash onset) should be extended until all of his symptoms resolved. Reports of measles in other immunocompromised patients have documented prolonged viral shedding [12,13].

We began contacting the school board and one of the physician’s offices that the child had attended to gather the list of exposed contacts that needed to be excluded based on our usual procedure for wild-type measles. Subsequently, after discussing the strong likelihood that this child had vaccine-associated measles with the pediatric infectious disease specialist, we decided to change our exclusion criteria. Although we had still not received the genotyping results to indicate that this was a vaccine-associated measles case, we proceeded on the assumption that it was the most likely cause.

We assumed that all immunized individuals and those with naturally-acquired infection were immune and would not be excluded. Unimmunized immune competent individuals were not considered to be susceptible because transmission of vaccine-associated measles had not been documented in this group. However, we were concerned that immunocompromised contacts could develop vaccine-associated measles and decided to exclude these individuals for the duration of the incubation period, in the event they developed symptoms of vaccine-associated measles similar to the index case. Since our patient was immunocompromised and had symptoms of classic measles, we postulated he could be more infectious than other cases of vaccine-associated measles. By the time we were notified of the case, the time window to offer immune globulin (Ig) had passed and we could not offer this to immunocompromised contacts.

We notified all contacts by sending a letter to watch for symptoms of measles and to seek medical attention if symptoms developed. We also indicated that those with immunocompromising conditions (e.g. pregnancy, immunosuppressive therapy, immunodeficiency) were at higher risk of acquiring measles and for developing more severe disease and asked these people to call Peel Public Health for further advice. We did not receive calls from any contacts reporting they were immunocompromised and no secondary cases were reported. Subsequent genotyping confirmed this case was caused by vaccine strain measles virus.

### Discussion

Vaccine-associated measles cases are rare and only one clinical case report of possible transmission has been published to our knowledge [14], however this case of potential transmission was based on a clinical diagnosis and was not microbiologically confirmed to be due to vaccine strain measles virus. It is uncertain if cases have not been reported because vaccine-associated measles strains are not transmissible or because of extremely low infectivity. Alternatively, susceptible contacts of these cases could have been infected, but were not detected because they had subclinical disease. Vaccine-associated mumps in close contacts of children who had received primary vaccination with MMR have been reported [3] so transmission of vaccine-associated measles seemed theoretically possible.

Canada is in the process of documenting measles elimination and the majority of measles cases in Canada are now imported from measles endemic countries [15]. However, in this case, the child did not have a travel history or exposure to a known measles case. Given the very high likelihood that this case was caused by vaccine strain measles virus (even prior to laboratory confirmation) we decided not to exclude all contacts from the usual high-risk settings (e.g. school, health care settings or workplaces). We stratified contacts into immune competent and compromised groups and decided to exclude the latter group conservatively as if they were exposed to wild-type virus because the risk of transmission of vaccine-associated measles from an immunocompromised case in this population, while clearly very low, theoretically may not be zero. No exclusions were applied to immunocompetent contacts.

Given the theoretical risk of an immunocompromised contact developing vaccine-associated measles, we decided to send a letter to all contacts advising them to watch for symptoms of measles and indicated that immunocompromised individuals should call our public health department. Self-identified immunocompromised contacts would be assessed for susceptibility and excluded from high-risk settings if required, similar to our procedure for wild-type measles. We could not offer Ig for these contacts since the six-day post-exposure window period had passed. If Ig had been a viable option, this could have been individually considered for immunocompromised contacts. However, we did not receive any calls and there were no secondary cases.

This case supports the belief that vaccine-associated measles is of very minimal risk to contacts, regardless of immune status. Indeed, current recommendations from national immunization advisory bodies, recommend that close contacts of immunocompromised patients should be vaccinated with measles vaccine to protect this vulnerable group [15,16]. Rashes after MMR vaccination occurs in 5% of vaccinees [16] and public health follow-up of vaccine-associated measles is not necessary [11].

Wild-type measles is one of the most infectious diseases known with a basic reproductive number ([Ro] the expected number of secondary cases due to an index case in a susceptible population), estimated to be up to 18 [17]. However, our immunocompromised patient with documented vaccine strain measles exposed numerous people with no reported virus transmission. Nonetheless, the true risk to immunocompromised patients from a case who is also immunocompromised is unknown and may not be zero. For immunocompromised contacts of vaccine-associated measles clinically indistinguishable from wild-type measles in an immunocompromised patient, we suggest managing these contacts as if they have been exposed to wild-type measles. Measles vaccination in immunocompromised patients is generally contraindicated because of the risk of disseminated measles vaccine virus infection. For this reason, we wanted to advise immunocompromised contacts to monitor for measles symptoms and exclude them from high risk settings.

## Conclusions

This case report describes the contact tracing experience of our public health department in the management of a vaccine-associated measles case in an immunocompromised child. Given the uncertainty in transmissibility of vaccine virus from an immunocompromised case exhibiting classic measles symptoms to immunocompromised contacts, we decided to exclude this high risk group since transmission of vaccine-associated measles to this population was likely very low, but theoretically possible. There were no secondary cases reported, confirming that transmission risk is very low. Although the risk appears negligible, exclusion and administration of Ig may be considered for susceptible, immunocompromised contacts of cases of vaccine associated measles in immunocompromised individuals.

### Consent

Written informed consent was obtained from the parents of the patient for publication of this case report. A copy of the written consent is available for review by the Series Editor of this journal.

## Abbreviations

HSCT: Hematopoetic stem cell transplant; HSV-1: Herpes simplex-1; Ig: Immune globulin; MMR: Measles, mumps, rubella; NP: Nasopharyngeal; PCR: Polymerase chain reaction; rRT-PCR: Real-time reverse transcriptase polymerase chain reaction.

## Competing interests

The authors declare that they have no competing interests.

## Authors’ contributions

MH conceptualized the case study and took the lead in drafting the manuscript. KLS provided the clinical details of the case study, conducted a literature review and participated in drafting the manuscript. CF and IM provided the public health details of the case study and critically reviewed the manuscript. JBG, AS, JH acquired the microbiological data for the case study and critically reviewed the manuscript. SLD reviewed the literature and critically reviewed the manuscript. SKM conceptualized the case study, provided supervision, participated in the literature review and in drafting the manuscript. All authors read and approved the final manuscript.

## Pre-publication history

The pre-publication history for this paper can be accessed here:

http://www.biomedcentral.com/1471-2458/13/269/prepub
